# Written reminders increase vaccine coverage in Danish children - evaluation of a nationwide intervention using The Danish Vaccination Register, 2014 to 2015

**DOI:** 10.2807/1560-7917.ES.2017.22.17.30522

**Published:** 2017-04-27

**Authors:** Camilla Hiul Suppli, Mette Rasmussen, Palle Valentiner-Branth, Kåre Mølbak, Tyra Grove Krause

**Affiliations:** 1 Department of Infectious Disease Epidemiology and Prevention, Statens Serum Institut, Copenhagen, Denmark; 2 National Institute of Public Health, University of Southern Denmark, Copenhagen, Denmark

**Keywords:** Vaccination Coverage, Immunisation Information System, reminders, epidemiology, vaccines and immunisation, vaccine-preventable diseases

## Abstract

We evaluated a national intervention of sending written reminders to parents of children lacking childhood vaccinations, using the Danish Vaccination Register (DDV). The intervention cohort included the full birth cohort of 124,189 children born in Denmark who reached the age of 2 and 6.5 years from 15 May 2014 to 14 May 2015. The reference cohort comprised 124,427 children who reached the age of 2 and 6.5 years from 15 May 2013 to 14 May 2014. Vaccination coverage was higher in the intervention cohort at 2.5 and 7 years of age. The differences were most pronounced for the second dose of the measles-mumps-rubella vaccine (MMR2) and the diphtheria-tetanus-pertussis-polio vaccine DTaP-IPV4 among the 7-year-olds, with 5.0 percentage points (95% confidence interval (CI): 4.5–5.4) and 6.4 percentage points (95% CI: 6.0–6.9), respectively. Among the 2.5 and 7-year-olds, the proportion of vaccinations in the preceding 6 months was 46% and three times higher, respectively, in the intervention cohort than the reference cohort. This study indicates a marked effect of personalised written reminders, highest for the vaccines given later in the schedule in the older cohort. In addition, the reminders increased awareness about correct registration of vaccinations in DDV.

## Introduction

Immunisation is one of the most successful and cost-effective [1] primary prevention tools in both low- [2] and high-income settings [3]. It is a public health priority to obtain a high vaccination coverage in the population to reduce the burden of vaccine-preventable diseases (VPD) [4]. However, in many high-income countries, the coverage rates are still below the target levels established by international [4] and national advisory committees [5]. The ongoing transmission of measles in Europe shows the consequences of the low coverage rates [6,7], missing the World Health Organization (WHO) goal of elimination [8].

In Denmark, all recommended childhood vaccinations are administered free of charge by the general practitioners (GPs). Still, Danish coverage rates for the second measles-mumps-rubella vaccination (MMR2) and the diphtheria-tetanus-pertussis-polio (DTaP-IPV4) booster are currently below 90%. Several risk factors for missing childhood vaccination have been identified, and parents forgetting the vaccination is one of the most frequent causes [9,10]. In Denmark, the GPs do not routinely remind parents about missing vaccinations.

A Cochrane review from 2005 of patient-reminder studies in Australia, Canada, Denmark, New Zealand, the United Kingdom and the United States (US) concluded that reminder and recall interventions increased the proportion of children being vaccinated [11]. Current evidence supports the use of postal reminders as part of a standard management of childhood immunisations [12].

The recommended childhood vaccination schedule for children from birth to five years is shown in Table 1 [13].

**Table 1:** The Danish childhood vaccination schedule up to five years of age, 2008–2017

Age	Vaccination
3 months	DTaPHib-IPV1 + PCV1
5 months	DTaPHib-IPV2 + PCV2
12 months	DTaPHib-IPV3 + PCV3
15 months	MMR1
4 years	MMR2
5 years	DTaP-IPV4

Vaccines are offered free of charge and administered by general practitioners. Reminders were issued for all vaccines except Hib and PCV.

DTaPHib-IPV: vaccine against diphtheria, tetanus, acellular pertussis, *Haemophilus influenzae* b, inactivated poliovirus; MMR: measles-mumps-rubella vaccine; PCV: pneumococcal conjugate vaccine.

In Denmark, a national electronic Immunisation Information System (the Danish Vaccination Register (DDV)) contains information on all vaccinations given in the childhood vaccination programme since 1996 [14]. Linkage of data from DDV with other administrative registers using the Danish personal identification number provides a unique opportunity to implement a national intervention aimed at parents of children with missing vaccinations.

In 2014, a change in the health legislation allowed Statens Serum Institut (SSI) to use the DDV to send written reminders to parents with missing childhood vaccinations at 2, at 6,5 and at 14 years of age [15]. Implementation of the intervention began on 14 May 2014. The time points for sending out reminders were selected based on the Danish childhood vaccination schedule and manageable administrative practices [16].

The aim of the present study was to assess the effect of in the first year of this nation-wide intervention on the vaccination coverage in Denmark.

## Methods

### Design and population

This study focused on an intervention caused by a policy change. The intervention cohort comprised children who turned 2 and 6.5 years and lived in Denmark between 15 May 2014 and 14 May 2015 and the reference cohort comprised children who turned 2 and 6.5 years of age between 15 May 2013 and 14 May 2014, the year before the national intervention was implemented. The vaccination coverage by vaccine type was compared for the intervention and the reference cohort at 2.5 and 7 years of age, so that the follow-up period was 6 months for every child in the study.

### Civil registry system

In Denmark, all residents are assigned a unique personal identification number (CPR number) that is recorded in the civil registry system. The system includes information on date and place of birth, date of immigration and emigration, previous and present place of residence and links to family relations including siblings, parents, and parent custody information. The birth cohorts were identified in the civil registry system.

### The Danish Vaccination Register

The Danish Vaccination Register (DDV) is a national immunisation system comprising all citizens in Denmark. The DDV contains information on all vaccinations given in the childhood vaccination programme from 1996 onwards, including the CPR number of the recipient, date of vaccination, name and Anatomical Therapeutic Chemical (ATC) classification group of the vaccine and identification on the vaccine provider. In Denmark, vaccines are administered by general practioners (GPs). Since 2013, citizens have had online access to their own and their children’s vaccinations status and health professionals can access the patients’ vaccination status. In case registration of previous vaccinations was missed, both patients and doctors can register historical vaccinations online. Data registered by parents are validated by the GP [14]. Effective from 15 November 2015, real-time reporting of all vaccinations administered by medical doctors and their assistants has become mandatory.

### The reminder database

The reminder system was implemented on 15 May 2014. All children who turn 2, 6.5 and 14 years lacking at least one vaccination in the childhood vaccination programme are identified in the DDV. Reminders concern all vaccinations in the childhood programme except for the pneumococcal conjugate vaccine, which is not recommended in the Danish childhood vaccination programme for children older than 2 years, and the *Haemophilus influenzae* b vaccine, which is combined with the DTaP-IPV and recommended for children younger than 6 years. For 2- and 6.5-year-old children, the reminder is sent to the parent in custody of the child. If the parents have joint custody but do not share the same address, the reminder is sent to both parents [16]. Information on all written reminders is saved in a database.

### Main outcome measures

The number of administered vaccinations was calculated on an individual level as the number of vaccines in the ATC groups used in the Danish childhood vaccination programme that contain MMR (J07BD52, J07BD53, J07BD54, J07BD01 and J07BE01) and DT (J07AF01, J07CA06, J07CA09, J07CA11, J07CA02, J07CA12, J07AJ52 and J07CA01). Timing of vaccinations and minimum intervals between vaccinations were not taken into account.

To compare the time–response relationship between receiving reminders and registered vaccinations in the intervention with the reference cohort, the numbers of vaccines registered where calculated for a 6-month period after the children turned 2 and 6.5 years.

### Statistical methods

Data analysis was conducted using Stata (Version 12, StataCorp, College Station, Texas) and SAS software version 9.4 (SAS Institute Inc., Cary).

For each cohort, vaccination coverage was calculated as the percentage of children alive and living in Denmark who had received a vaccine registered in the DDV. We counted all vaccines without considering the reported dose, as the dose number is often incorrect. The change in vaccination coverage after receiving a written reminder was calculated as the differences in vaccination coverage between the intervention and the reference cohorts among children aged 2.5 and 7 years (vaccine coverage difference measured in percentage points).

### Ethical considerations

The study was purely register-based and was notified to the Danish Data Protection Agency with record number 2008–54–0474. Parents of children who were not fully vaccinated were contacted as part of the intervention [15].

## Results

A total of 124,189 children were included in the intervention cohort. In the study period, reminder letters were sent to parents of 43,288 children (22,621 boys and 20,667 girls). Among the 2-year-olds, 27% (15,628/57,770) missed at least one vaccination and among the 6.5-year-olds, it was 42% (27,660/66,419). For 6,970 children, letters were sent to two parents because of divorce and joint child custody. A total of 423 letters were returned to sender unopened, and 56 parents made a request by either letter or encrypted email to opt out of the vaccination notification service.

In general, the vaccination coverage was highest for the first vaccinations in the schedule, with coverages reaching 90% and above for the two first DTaP-IPV vaccines, and the largest effect of the reminder letters was seen among the older children, Table 2. For the 2.5-year-olds, we saw for all vaccines in the intervention group a statistically significantly higher vaccination coverage of 1–2 percentage points for DTaP-IPV and 3.9 for MMR1. For the 7-year-olds, the difference was most pronounced for MMR2 and DTaP-IPV4: 5.0 percentage points (95% confidence interval: 4.5–5.4) and 6.4 percentage points (95% CI: 6.0–6.9), respectively. Stratifying by sex did not change the estimates.

**Table 2:** Vaccination coverage assessed at ages 2.5 and 7 years, and risk difference by vaccine dose in reference cohort (n = 124,427) vs intervention cohort (n = 124,189), Denmark, 15 May 2013–14 May 2015

Number of vaccine doses received	2.5 years					7 years				
	Reference (2013–14) N = 58,943	Vaccination coverage	Intervention (2014–15) N = 57,770	Vaccination coverage	VCD (95% CI)	Reference (2013–14) N = 65,484	Vaccination coverage	Intervention (2014–15) N = 66,419	Vaccination coverage	VCD (95% CI)
	n	%	n	%	%	n	%	n	%	%
DTaP-IPV 1	56,890	96.5	56,337	97.5	1.0 (0.8–1.2)	63,555	97.1	64,996	97.9	0.8 (0.6–1.0)
DTaP-IPV 2	55,759	94.6	55,210	95.6	1.0 (0.7–1.2)	61,793	94.4	63,308	95.3	1.0 (0.7–1.2)
DTaP-IPV 3	49,869	84.6	50,019	86.6	2.0 (1.6–2.4)	58,562	89.4	60,806	91.5	2.1 (1.8–2.4)
MMR1	50,640	85.9	51,879	89.8	3.9 (3.5–4.3)	61,684	94.2	63,525	95.6	1.4 (1.2–1.7)
MMR2	NA	NA	NA	NA	NA	50,108	76.5	54,133	81.5	5.0 (4.5–5.4)
DTaP-IPV 4	NA	NA	NA	NA	NA	44,556	68.0	49,462	74.5	6.4 (6.0–6.9)

CI: confidence intervals; DTaP-IPV: diphtheria-tetanus-acellular pertussis-inactivated poliovirus vaccine; MMR: measles-mumps,-rubella vaccine. NA: not applicable; VCD: vaccination coverage difference.

We calculated the ratio of children who received the vaccination per number of children in the birth cohort, alive and living in Denmark. Coverage was assessed at 6 months after the child turned 2 or 6.5 years in the period from 15 May 2014 to 14 May 2015 for both the reminder and the intervention cohort. The vaccination coverage by vaccine type was compared for the intervention and the reference cohort at 2.5 and 7 years of age, so that the follow-up period was 6 months for every child in the study.

### Vaccine registration in a 6-month follow-up period

Table 3 displays the number of vaccines registered in the 6-month period after the child turned 2 or 6.5 years for the intervention and reference cohorts. Overall, among the 2.5-year-olds, the proportion of registered vaccines in the 6-month follow-up period was 46% higher in the intervention cohort compared with the reference cohort. In the 7-year-olds, this proportion was three times higher in the intervention compared with the reference cohort. 

**Table 3:** Vaccine doses registered in the 6-month follow-up at ages 2 and 6.5 in reference (n = 124,427) vs intervention cohort (n = 124,189), and the ratio (intervention vs reference), Denmark, 15 May 2013–14 May 2015

Number of vaccine doses received in 6 month	2.5 years			7 years		
	Reference (2013–14) N = 58,943	Intervention (2014–15) N = 57,770	Ratio (95% CI)	Reference (2013–014) N = 65,484	Intervention (2014–015) N = 66,419	Ratio (95% CI)
	Δn	Δn		Δn	Δn	
DTaP-IPV 1	61	155	2.59 (1.93–3.48)	95	247	2.56 (2.02–3.24)
DTaP-IPV 2	123	289	2.39 (1.94–2.95)	57	241	4.16 (3.12–5.55)
DTaP-IPV 3	642	1,319	2.07 (1.89–2.28)	222	745	3.28 (2.83–3.81)
MMR1	3,884	5,211	1.34 (1.29–1.39)	416	523	1.24 (1.09–1.41)
MMR2	NA	NA	NA	558	2,405	4.14 (3.77–4.53)
DTaP-IPV 4	NA	NA	NA	952	3,926	3.89 (3.63–4.18)
Total	4,710	6,974	1.46 (1.41–1.51)	2,300	8,087	3.20 (3.06–3.35)

CI: confidence intervals; DTaP-IPV: diphtheria-tetanus-acellular pertussis-inactivated poliovirus vaccine; MMR: measles-mumps,-rubella vaccine. NA: not applicable.

We calculated Δn as the number of vaccines administered during the study period, and the ratio of intervention vs reference cohort.

In the intervention cohorts, 15,061 DTaP-IPV and MMR vaccines were registered in the following 6 months. In the reference cohort a markedly smaller number of 7,010 vaccines where registered in the 6-month period. Most vaccines were administered within the follow-up period, but 9.3% of vaccines among 2-year-olds and 20.7% among 6.5-year-olds where registered directly in the DDV by a doctor or a parent with a vaccination date that was before receiving the reminder.

Table 4 shows the total number of vaccines registered in the 6-month follow-up period. Of these, some were previously given vaccines now registered by citizens or doctors in the DDV. In the intervention cohort, a proportion of vaccines (9% and 21%) had been administered before the intervention and reflects registration of historic vaccinations that had not previously been registered in the DDV. This was mainly seen for the intervention cohort because information on the possibility to register historic vaccinations was included in the reminder letter.

**Table 4:** Vaccines registered by parents or doctors in the Danish Vaccination Register in the 6 month follow-up period in the intervention and reference cohorts, Denmark, 15 May 2013–14 May 2014 (reference) and 15 May 2014–14 May 2015 (intervention)

	Total number of vaccines registered in 6 months after reminder date	Vaccines registered by parents with vaccination date before reminder date	Vaccines registered by GP´s with vaccination date before reminder date	Delayed vaccine registration
	N	n	n	%
2.5 years				
Intervention cohort	6,974	487	159	9.3
Reference cohort	4,710	0	3	0.1
7 years				
Intervention cohort	8,087	1,323	354	20.7
Reference cohort	2,300	0	0	0.0

GP: general practitioner.

## Discussion

A simple intervention of sending out written reminders increased the vaccination coverage. The effect was higher for the children at 6.5 years than 2 year of age and the strongest effect was observed for the DTaP-IPV4 vaccine. Here, the coverage was 6.4 percentage points higher in the intervention group than in the reference group. The increase in coverage was mostly due to administration of vaccines but also due to increased compliance with registering previously given vaccines in the DDV. This study showed that it is feasible to use a national immunisation register for sending out written reminders to parents of children who lack vaccines in the childhood vaccination programme.

The uptake was highest for the first vaccines in the schedule. Participation in well-child visits decreases after the child has turned 1 year and parents may therefore not be reminded of the importance of adhering to the childhood vaccination programme. In addition, parents’ lack of time after having re-entered work after the 12-month parent leave customary in Denmark may have an impact. During maternal/paternal leave there is higher flexibility for immunisation appointments without having to accommodate a work schedule [9]. Another hypothesis is that parents may assume that a delayed vaccination cannot be caught up after a substantial period of time has passed and therefore disregard the importance of fulfilling a vaccination series. An official reminder may affect this notion.

Among the two and 6.5-year-olds in the intervention groups, we saw a significantly increased coverage 6 months after the intervention for all the vaccines. The increase was most pronounced for the two latest vaccines in the schedule. A possible explanation why the effect in the youngest age group was smaller could be that the children lacking vaccinations are delayed in their schedule and would have received the vaccinations later, regardless of the reminder. These results indicate that it may be inefficient to distribute reminders close to the scheduled date for each vaccine and that the optimal age in this study was in the older children. The larger number of vaccines registered in the intervention cohort in the first 6 months after reminder, compared with the reference cohort, supports the fact that the effect we experience is an actual increase in vaccines administered and not just a persistent difference between the two cohorts.

In an era of increasing complexity of immunisation schedules, it is important to understand and promote interventions that work. Studies have shown that patient reminder and recall systems in primary care settings are effective in improving immunisation rates in developed countries [11]. Our findings support the review by Harvey et al. who concluded that postal reminders are an effective measure [12]. Our national reminder system based on data linkage with an immunisation register is, to our knowledge, unique to Denmark, although immunisation registers are currently in place or under development in several other countries.

Beyond improving immunisation rates, reminders have additional benefits for the patient and practice. Studies have shown that patients who do not comply with immunisation programmes are likely not to comply with other measures of preventive care either [17], and that immunisation reminder or recall systems also improve other preventive care measures [18].

A study from 2007 showed that forgetfulness or oversight was the most frequent explanation for missing vaccines in Danish children [9]. A written reminder intervention may target this group of parents, while this type of intervention will not be effective in parents who do not want to vaccinate their children. The finding of the present study corroborates that the majority of lacking vaccines is explained by oversight [9,10,19]. We experienced only a small number of parents who actively opted out of the programme (n = 56, corresponding to a frequency of 0.001%), but this does of course not take into consideration parents ignoring the reminder letter. In agreement with previous findings in Denmark [9], Smith et al. found in a US study that reasons other than negative vaccine-related beliefs accounted for the vast majority of unvaccinated children and adolescents [20].

### Strength and limitations

To our knowledge, this is the first evaluation of a written reminder service based on a national immunisation information system that covered complete birth cohorts. The study was large and nationwide and included all Danish children who turned 2 and 6.5 years in a 2-year period. The major strength of our study was the use of individual data from population-based registries covering the total population with complete follow-up. Further, children included in the analyses were from adjacent birth cohorts, minimising the risk of bias do to time-dependent factors.

Childhood vaccinations reimbursed by the Danish healthcare regions to the GPs are registered in the DDV. We recognise that there may be missing vaccine registrations. In 2013, Wójcik et al. validated the Danish childhood vaccination database in regards to the coverage of the DTaP-IPV4 and identified under-reporting estimated to 3–4 percentage points, mainly due to GPs not registering given vaccinations [10]. Our findings that at least 15% of the registered vaccinations in the follow-up period were administered before the reminder was received can be regarded as a measure of under-registration in the DDV. An additional positive effect of the reminder intervention was raised awareness about registration of vaccinations among GPs and the general population and led to an improvement of the immunisation register data.

For calculation of vaccination coverage, we counted all vaccines without considering the reported dose, as the dose number is often incorrect. In case of missing registrations of vaccines, this method leads to a ‘false’ low coverage for the later vaccines in a series and possibly an underestimation of the effect of the reminder on the first vaccines in a series. The false low coverage is, however, the case for both the intervention and reference cohorts. The vaccine type-specific vaccination coverages presented in this study may differ from nationally reported coverage data, which calculates coverage including dose code.

We are not aware of recommendations or previous studies on the most appropriate time for follow- up after sending written reminders. As it may take some time from receipt of reminder until the parents make a vaccination appointment, we evaluated a possible effect after six months.

Natural experimental studies enable us to study effects in a whole population or, in this case, a whole subgroup of the population [21]. Due to the applied design of a reference consisting of a pre-intervention cohort, any time-dependent factors affecting the coverage may complicate conclusions of causality. A randomised controlled trial would have been the strongest evaluation strategy, but the nature of the nationwide policy change evaluated in this study precluded this possibility. We cannot rule out the possibility that differences in our intervention cohort and the previous year’s cohort could be due to other factors, as we cannot fully disclose all possible measures of effect on the vaccination coverage. It is possible that the media attention given to measles outbreaks as in the US [22,23] and in Germany [6,24] had a positive influence on the awareness of communicable infectious diseases and subsequently on the vaccination coverage in the childhood vaccination programmes. However, this attention should have had a similar impact on both the intervention and reference groups. On the other hand, there has been a heated parallel debate in the Danish media on perceived adverse events to the human papillomavirus vaccine and a dramatic drop in HPV vaccination coverages among 12–14-year-old girls in Denmark, with only 16% of girls born in 2003 finishing the vaccination programme compared with 79% of girls born in 2000 [25]. This may have affected the vaccination coverage of other childhood vaccinations negatively. In addition, several healthcare regions in Denmark have implemented different reminder systems where GPs are informed about unvaccinated children connected to their practice. However, to our knowledge, these interventions have been the same for the two cohorts and we therefore assume that they did not seriously affect the interpretation of our results. That more vaccines were administered in the follow-up period in the intervention cohort than in the reference cohort leads us to believe that this was true effect of the intervention.

As the reminders were send out as a national policy, various stakeholders reviewed the wording, and due to legal constraints, it is currently not possible to test wording in an RCT approach. Therefore, the effect of any changes in the reminder system can only be followed in the target groups at the national level.

Our data were generated by sending out letters to parents of a 43,288 children registered with missing vaccines in the DDV. Denmark is a country with a high level of both interpersonal trust and trust in the authorities [26] and it is therefore plausible that a large percentage of the letters where in fact opened and acted on, which is not necessarily true for other countries with lower levels of trust.

### Implications

A reminder is just one of the tools that can be used to raise the coverage. The WHO Regional Office for Europe has developed The Guide to Tailoring Immunisation Programmes (TIP) which aims to provide methods and tools to identify susceptible populations, determine barriers to vaccination and implement evidence-based interventions [27]. This approach has already been applied in Sweden [28].

The current intervention only comprised sending written catch-up reminders to certain age groups. We have not reached the target vaccination coverage and could have hoped for a better response. More research is needed to understand how wording, format, timing of sending out the reminders as well as resending reminders affect the response. However, it is clear from the current study that reminders cannot stand alone in the efforts to increase vaccination coverage. On the other hand, the costs of the reminder system have been low, with DKK 1.7 million (EUR 229,000) in the developmental stage and a yearly operational cost of DKK 1 million (EUR 134,000). Since November 2016, we have been sending electronic reminders at the same time points as described in the study, which is an even cheaper solution. How this will affect the coverage is of great importance and must be evaluated in the coming years. Technically, it would also be feasible to send out electronic reminders both before and after scheduled vaccination, and more advanced reminder systems are currently under development. Odone et al. concluded that although the use of other communication channels such as websites and mobile phone apps has great potential, the data are scant for now [29]. The change to mandatory registration of vaccines by doctors may lead to more timely and complete registration in the register, which also needs to be further evaluated. Several countries are currently developing IIS and may have an opportunity to implement similar reminder interventions. However, the effect may be influenced by cultural settings and organizational practices that differ from country to country.

## Conclusions

Our evaluation showed that written reminders increased vaccination coverage. The reminders are also likely to have an indirect effect by increasing awareness about correct registration of vaccines in the immunisation register. Immunisation registers have already proven extremely useful in providing reliable information on vaccination coverage and supporting studies on vaccine effectiveness and safety. The study presented here showed that the immunisation register can also be used for reminder services, which may have the potential to improve coverage in national vaccination programmes.
